# Clean Label Trade-Offs: A Case Study of Plain Yogurt

**DOI:** 10.3389/fnut.2021.704473

**Published:** 2021-07-30

**Authors:** Sara Maruyama, Juyun Lim, Nadia A. Streletskaya

**Affiliations:** ^1^Department of Food Science and Technology, Oregon State University, Corvallis, OR, United States; ^2^Department of Applied Economics, Oregon State University, Corvallis, OR, United States

**Keywords:** clean label, choice experiment, ingredient list, consumer preferences, texture, price

## Abstract

Consumer demand for clean label has risen in recent years. However, clean label foods with simple and minimalistic ingredient lists are often expensive to produce and/or may possess less desirable sensory qualities. Accordingly, understanding consumer preferences regarding the clean label trend would be of great interest to the food industry. Here we investigate how ingredient lists and associated sensory quality descriptions may influence consumer preferences using a hypothetical choice experiment. In particular, we test the impacts of four common stabilizers (carrageenan, corn starch, milk protein concentrate, and pectin) and textural characteristics on preferences and willingness to pay for plain yogurt. A total of 250 yogurt consumers participated in the study. The results of a mixed logit analysis suggest that clean labeling significantly increases the likelihood of consumer choice, while poor texture reduces consumer choice. More importantly, the negative impact of poor texture seems to be less significant for clean label yogurts compared to that for yogurts with longer ingredient lists. Among all stabilizers, corn starch in particular has a significant negative impact on consumer choice. The estimated average consumer willingness to pay for clean labels is between $2.54 and $3.53 for 32 oz yogurt formulations. Furthermore, clean labels minimize the negative impact of textural defects with consumers willing to pay an estimated premium of $1.61 for the family size yogurt with a simple ingredient list. Results of latent class modeling reveal two classes of consumers with similar patterns of demand who prefer clean labels and, on average, would rather purchase a yogurt with a textural defect than opt out of purchasing a yogurt entirely. Implications for the food industry are discussed.

## Introduction

In recent years, consumers demand for specific dietary and nutritional characteristics in their foods [e.g., reduced sugar, free from artificial preservatives; (1)]. This shift in consumer preference has resulted in a strong push in the food industry to remove certain ingredients through reformulation (2). Common ingredients targeted for removal include those that are synthetically derived (e.g., Red 40, artificial flavors) and have long, “chemical-sounding” names [e.g., carrageenan, methyl crystalline cellulose; (3)]. Although these ingredients are deemed safe by regulatory agencies, they are perceived as harmful by consumers due to their lack of familiarity (4, 5) and risk perception of chemicals (6, 7). While many factors likely play roles in the demand for “clean label” foods, existing research suggests that health and sustainability concerns, for example, motivate consumers to seek such products (8–10). Additional research investigating the clean label trend suggests that consumers prefer short ingredient lists that contain familiar, minimally processed ingredients (11, 12). Accordingly, food companies have made great efforts in reformulating their products to achieve cleaner labels (2, 13).

The move away from highly-processed ingredients in the food industry can be seen as companies across the retail landscape strive to clean up their labels. In the United States, major food companies began cleaning up their ingredient lists around 2010 (3). For example, Hershey's began reformulating their products by replacing their sugar beet-derived sugar, a crop grown primarily from genetically modified (GMO) seeds, with sugar cane-derived sugar in 2015 (14). A year later, Campbell launched their clean label line of soups called “Well Yes!”, which contained no artificial flavors or colors, and/or modified starches (15). Today, cleaner labels are ubiquitous across multiple food categories, including bakery, soft drinks, snacks, prepared soups, and dairy products (16, 17).

Ingredient blacklists compiled by influential retailers, such as Kroger and Whole Foods, are one source of criteria for companies striving to develop cleaner labels (18). Common clean label reformulation effort involves either complete removal of undesirable ingredients or their replacement with more natural alternatives. The latter process is generally expensive and time-consuming as those ingredients are often more costly, and the resulting products often possess less desirable sensory characteristics compared to their original counterparts (19, 20). In particular, the ingredients that are considered undesirable by consumers are often designed or modified to maximize their functionality within a food. Thus, replacement of these ingredients (e.g., modified corn starch) with natural alternatives (e.g., native corn starch) can result in an increase in the ingredient usage rates, an increase production costs, and/or potentially poor sensory characteristics (21–24). The alternative of complete ingredient removal often has similar challenges (25).

Although consumers state a preference for cleaner labels, consumers' behaviors and actions sometimes contradict their preferences (26), especially when other factors are involved. Arguably, the two upmost important factors might be sensory characteristics and price of the product in question. Sensory attributes such as flavor, texture, and appearance are commonly identified as product characteristics of high importance to consumers (27–29). For example, consumers are unwilling to compromise “taste” for health benefits in functional foods (30, 31). Price is another factor that impacts purchase behavior. Streletskaya et al. (32) showed that price increases through taxes have the potential to reduce purchase of unhealthy foods leading to reduced intake of certain undesirable nutrients (i.e., calories, cholesterol, etc.). However, in some situations (particularly for higher income consumers) price may have less of an impact on demand for food compared to non-economic factors (33). Thus, while the costs to produce a clean label food increase relative to its original formulation and the increased costs are passed on to the consumer, it is unclear what premium consumers may be willing to pay for clean label foods. Furthermore, while consumers might, on average, have a higher willingness to pay for clean label products, this price premium might not be high enough to cover the costs of reformulation, similarly to premia and cost dynamics of organic foods (34, 35). The tradeoffs between label cleanliness, sensory characteristics, and price are of particular interest to companies considering reformulation, as it is unclear how these factors influence each other.

Yogurt is a food product category where significant reformulation efforts have been made to satisfy consumer demand for clean label (36). Reformulation efforts have targeted eliminating ingredients such as artificial coloring agents, chemical preservatives, and modified starches (37–40). For sensory characteristics, creamy mouthfeel, and smooth appearance seem to be critical in yogurt (41, 42), along with a lack of or minimal syneresis [i.e., expulsion of liquid whey from the yogurt (white mass); (43–46)]. To achieve such sensory characteristics, stabilizers and thickeners are commonly used in yogurt products (47). Most common food stabilizers and thickeners are various polysaccharides (48) such as carrageenan, corn starch, and pectin. Milk and whey protein concentrate are also commonly used in yogurt because they can add higher protein content while modulating thickness (49) and improving texture (50). Despite the common usage of stabilizers and thickeners in food, research has shown that consumers are not particularly knowledgeable about them (51). More importantly, recent study suggests that stabilizers and thickening agents are perceived as generally unnatural by yogurt consumers, compared to other ingredient categories such as sugars, preservatives, and coloring agents (52). Consequently, the food industry has put forth great efforts to replace highly processed stabilizers or to remove them entirely from their products (17, 53, 54).

The overarching goal of this study is to look at consumer demand for clean label while considering related sensory characteristics and price changes. To achieve this goal, we employ a hypothetical choice experiment, which is commonly used to examine how product characteristics affect consumer product choice. When defining “clean label” for the purpose of this study, we focused on four different stabilizers/thickening agents—carrageenan, corn starch, milk protein concentrate (MPC), and pectin—that are commonly used in yogurt manufacturing. We have created different ingredient lists for plain yogurts that range from just cultured pasteurized milk to yogurts that also included all four stabilizers and formulations in between^1^. Of note, the stabilizers used in this study were selected based on the results of a recent survey conducted by Maruyama et al. (52), ranging from relatively natural (pectin, corn starch) to relatively unnatural (MPC, carrageenan). As our study examines the potential tradeoffs consumers might be willing to make between different yogurt characteristics, sensory characteristics and price were considered as other key factors.

## Materials and Methods

### Consumers

Plain yogurt consumers were recruited through an existing pool of consumers from the Center for Sensory and Consumer Behavior. Additionally, flyers advertising the study were posted around the campus and electronic advertisements were sent though a university email newsletter. In order to qualify for the study, all respondents were required to fill out a screening survey. The inclusion criteria for the study participation were: (1) fluency in written and oral English, (2) age between 18 and 65 years, (3) a consumer of family-sized tubs of plain yogurt at a frequency of at least once every other week, and (4) not employed or involved in the food and beverage industry. The last criterion was set to ensure that only the responses of lay consumers were captured. Qualified respondents were invited to participate in the study. Written consent was obtained from each respondent prior to participation in the study. A total of 629 consumers expressed interest in our study and filled out the screening survey. Of those interested, only 336 met the inclusion criteria outlined above and a total of 250 consumers participated in the choice experiment due to scheduling availability. The study protocol was approved by the university's institutional review board (IRB-2019-0187).

### Research Design

#### Ingredient List

The ingredient lists of yogurts on the market were reviewed to determine which stabilizers were used in yogurt formulations. Additionally, industry experts in yogurt manufacturing were consulted. Four different stabilizers/thickening agents were chosen as ingredients of interest for this study: carrageenan, corn starch, MPC, and pectin. Carrageenan is derived from a type of red seaweed called Irish moss (55) and commonly used in synergy with other stabilizers to prevent syneresis and improve texture (56). While previous research (57–60) has questioned the safety and toxicity status of carrageenan, numerous reviews (61–64) and studies (65–67) support carrageenan's safety status. Corn starch, a starch-based polysaccharide, is a common household ingredient that is used to thicken soups, sauces, and fruit preparations. In general, native starches are considered to be clean label by researchers (68–70). MPC is a milk-derived ingredient obtained through membrane filtration of fluid milk. Similar to corn starch, it increases viscosity and minimizes syneresis in yogurt (71). In addition to its functionality as a stabilizer and thickener, MPC also serves as a means of protein fortification making it appealing to manufacturers looking to market high protein yogurts (71). Pectin is well-known in the food industry as a gelling agent and it functions as a stabilizer in yogurt and acidified dairy beverages (72). In dairy applications, it can be used as a stand-alone stabilizer or in combination with other stabilizers or thickeners. It is commercially derived from the cell walls of plants and primarily sourced from apples and citrus fruit (73). From a cost standpoint, pectin is more expensive than other stabilizers, but it is considered the stabilizer of choice for yogurts positioned as natural (74).

#### Texture Characteristics

Two possible texture characteristics were given as choices: overall good texture, or a texture with defects. A yogurt free of textural defects was described as, “Consumers generally perceive the texture of this yogurt to be good and free of defects (e.g., smooth, creamy),” while a yogurt possessing some defects was described as, “Consumers generally perceive the texture of this yogurt to have some defects (e.g., grainy, lumpy).”

#### Price

The yogurts in the hypothetical choice experiment were offered at $2.99, $4.49^2^, and $7.36 per 32 oz family size tub. These price levels were based off the retail prices of commercially available yogurts found in local grocery stores.

### Choice Experiment

Experimental design is based off standard practice (75) using a D-efficient design for a generic unlabeled format generated by Stata's create package. To minimize the cognitive burden on our respondents, a block design was incorporated into our experiment. One of two blocks, each with 11 choice sets, was randomly presented to the respondent, as described below.

**Figure 1:**
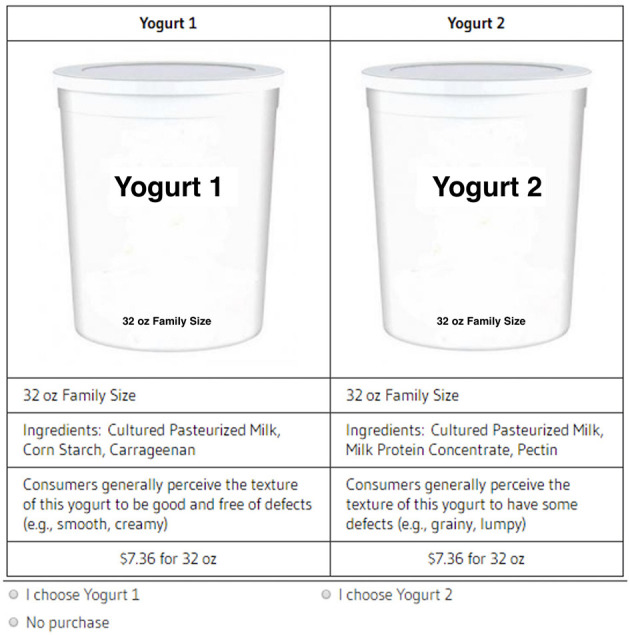
Choice set example.

Each participant was invited to attend one 25-min moderated session on campus. A cheap talk script was delivered at the start of the experiment, highlighting that even though the choices made in the experiment were hypothetical, respondents should carefully consider their yogurt preferences and budget constraints. Cheap talk scripts have been shown to be an effective tool for mitigating hypothetical bias (76). Each choice set was presented to respondents individually, and respondents were instructed to consider each choice set on its own merits. All respondents made selections in 11 choice sets (see Figure 1 for an example of a choice set).

Following the choice experiment, the participants were directed to rate their agreement regarding a series of different statements on a 10-point scale anchored at 1 indicating a strong disagreement and 10 indicating a strong agreement with the statements presented. Specifically, respondents were asked, “When I purchase yogurt I care about [taste/flavor/texture, price, ingredient list, the presence of milk protein concentrate, the presence of pectin, the presence of corn starch, and the presence of carrageenan].” These statements were aimed to assess the importance of quality (taste/flavor/texture,) price, ingredients, and the presence of MPC, corn starch, pectin, and carrageenan. After rating the statements presented, respondents were instructed to fill out a brief socio-demographic questionnaire.

## Results

### Demographics

The general socio-demographic characteristics of our sample population are summarized in Table 1. The mean age of our sample was 39 years old (18–65 years old; *SD*: 13.44 years old); 76% were female; 79% were Caucasian; 59% reported annual household incomes below $75,000 and 24% reported incomes of $100,000 or greater. Thirty-seven percent of respondents held bachelor degrees, 32% held master's degrees, 9% held professional/doctoral degrees, and 21% of respondents held less than a bachelor degree. Sixty-four percent of respondents reported to live in 2–4 person households and 26% reported to grocery shop for children 18 years old or younger within their households. These consumer demographic characteristics are similar to those reported in peer-reviewed research examining yogurt consumers (34, 77–81). Lastly, 92% of our sample reported to be primary shoppers within their households. Primary shoppers were defined in our study as individuals who are responsible for at least half of all household grocery purchases.

**Table 1:** Demographic characteristics.

**Factor**	**Category**	***N***	**%**
Gender	Female	189	75.6
	Male	59	23.6
	Other/Prefer not to say	2	0.8
Race	White/Caucasian	197	78.8
	Asian/Indian subcontinent	22	8.8
	Hispanic/Latino	6	2.4
	African American	3	1.2
	Other	22	8.8
Education	High School Diploma or Equivalent	14	5.6
	Associate/Technical/Trade/Vocational	11	4.4
	Some College (no degree)	28	11.2
	Bachelor's degree	93	37.2
	Master's degree	81	32.4
	Professional/Doctorate degree	23	9.2
Household income	< $34.999	71	28.4
	$35,000–$49,999	37	14.8
	$50,000–$74,999	39	15.6
	$75,000–$99,999	42	16.8
	$100,000–$149,999	50	20.0
	$150,000–$199,999	8	3.2
	>$120,000	3	1.2
Household size	1	83	33.2
	2	87	34.8
	3	34	13.6
	4	39	15.6
	5	6	2.4
	6+	1	0.4
Shop for children	Yes	65	26.0
	No	185	74.0
Primary household shopper	Yes	231	92.4
	No	19	7.6

### Mean Statement Ratings

Mean ratings and standard errors of agreement to attribute-related statements are summarized in Table 2. On average, Quality, specified as flavor, texture, and/or appearance, was rated as the most important attribute, followed by Price, Ingredients, Corn Starch, Carrageenan, Milk Protein Concentrate, and Pectin.

**Table 2:** Mean ratings (SE) of agreement to attribute-related statements.

**Quality**	**Price**	**Ingredients**	**Corn starch**	**Carrageenan**	**Milk protein concentrate**	**Pectin**
9.044 (0.0923)	7.824 (0.126)	7.220 (0.161)	4.856 (0.204)	4.240 (0.185)	4.076 (0.173)	3.984 (0.170)

### Model and Estimation Results

#### Model

Following common practice, we use an alternative specific mixed logit model (82) to analyze the choice experiment responses with utility specified as:

(1) $$U_{njt}=\beta_{n}\prime x_{njt}+e_{njt},$$

where *x*_*njt*_ is the vector of observed choice attributes of alternative j in the choice set t, β_*n*_ is the vector of parameters of interest that is unobserved for each decision maker *n* and varies in the population with density *f*(β|θ*), where θ are the parameters of the distribution of β in the population, assumed to be triangular for the price of yogurt coefficient, and the random variable *e*_*njt*_ is random and independent and identically distributed (IID) extreme value type 1. The mixed logit probability takes the standard form when the density function of *f*(β) is continuous:

(2) $$P_{ni}=\int \left(\frac{e^{\beta^{\prime }\;x_{ni}}}{\sum_{j}e^{\beta^{\prime }\;x_{nj}}}\right)f(\beta )d\beta$$

We then assume a discrete mixing distribution, modifying (2) to reflect a latent class model. The choice probability for the latent class model takes the following form:

(3) $$P_{ni}=\sum_{m=1}^{M}s_{m}\left(\frac{e^{b_{m}{\;}^{\prime }\;x_{ni}}}{\sum_{i}e^{b_{m}{\;}^{\prime }\;x_{nj}}}\right),$$

where there are M segments and the share of population in segment *m* is *s*_*m*_. The latent class model enables segmentation of consumer choices into different “classes” according to preference patterns. The number of classes in the model was specified based on the AIC and BIC criteria comparison across models with up to four consumer classes, resulting in a two-class model being identified as the best fit.

While the mixed logit model with alternative specific constants provides a general characterization of consumer preferences and allows for parameter heterogeneity for price, the latent class logit model segments participants into distinct classes, allowing for additional insights into consumer demand for clean label yogurts. Both models are estimated using Stata 15 with standard errors clustered at participant level to accommodate the fact each participant made 11 choices.

In the following results section, estimated impacts of our parameters of interest will be reported as odds ratios, which is the exponentiated form of the parameter coefficients. Additionally, we can use coefficients from our logit models to estimate willingness to pay (WTP) for statistically significant parameters. WTP estimates are obtained by using the following formula:

(4) $$WTP_{attribute}=-\frac{\beta_{attribute}}{\beta_{price}},$$

where β_*atttribute*_ is the estimate or coefficient for significant parameters of interest (i.e., clean label, poor texture, etc.), and β_*price*_ is the estimated price coefficient.

#### Estimation Results

##### Alternative Specific Mixed Logit With Core Yogurt Attributes Model (Specification 1)

In our base model specification (Specification 1), a mixed logit was used to model the impact of price and individual ingredients (i.e., carrageenan, corn starch, etc.) on the likelihood of choosing a yogurt. The impact of each ingredient is modeled using dummy variables. Dummy variables are binary categorical explanatory variables, which take on a value of 1 if a specific condition is met (i.e., pectin is present, corn starch is present, etc.) and a 0 if otherwise (83). Results of Specification 1 reveal an odds ratio of 0.60 (*p* < 0.001) for the price parameter (see Table 3). Thus, holding all parameters constant, a one dollar increase in the price of a yogurt decreases the odds of purchasing a yogurt by 40%. This finding was expected as consumers would naturally opt for a less expensive yogurt if presented with two yogurts that are otherwise identical. When the impact of each ingredient is examined, we find that all ingredient-related odds ratios are significant and <1.0, indicating that each ingredient has a negative impact on the odds of choosing a yogurt, ceteris paribus. Specifically, holding all parameters constant, the odds ratios of purchasing a yogurt are 0.82 (18% reduction) if pectin is present, 0.60 (40% reduction) if carrageenan is present, 0.40 (60% reduction) of MPC is present, and 0.39 (61% reduction) if corn starch is present (*p* < 0.001). Furthermore, we can estimate how much consumers are willing to pay to avoid these ingredients in yogurt using willingness to pay (WTP) estimates which are displayed in Table 4. On average, we estimate that consumers are willing to pay $0.38 to avoid pectin, $0.97 to avoid carrageenan, $1.75 to avoid MPC, and $1.80 to avoid corn starch for the 32 oz family size plain yogurt.

**Table 3:** Alternative specific mixed logit model specifications, results with odds ratios.

**Parameter**	**Specification 1**	**Specification 2**	**Specification 3**	**Specification 4**
Price	0.60^***^ (−16.98)	0.64^***^ (−15.02)	0.58^***^ (−10.63)	0.63^***^ (−12.55)
Pectin	0.82^**^	0.96	0.95	1.11
	(−2.82)	(−0.51)	(−0.69)	(1.06)
MPC	0.40^***^	0.46^***^	0.99	0.89
	(−10.87)	(−9.42)	(−0.09)	(−1.12)
Carrageenan	0.60^***^	0.63^***^	0.83^*^	0.88
	(−7.58)	(−6.16)	(−2.33)	(−1.64)
Corn starch	0.39^***^	0.36^***^	0.49^***^	0.46^***^
	(−10.17)	(−11.40)	(−8.04)	(−8.64)
Clean label	–	–	6.78^***^	3.31^***^
			(8.65)	(5.34)
Poor texture	–	0.44^***^	0.29^***^	0.26^***^
		(−10.13)	(−9.09)	(−11.55)
Clean label × poor texture	–	–	–	2.16^***^
				(4.82)
*Price triangular spread*	0.0095	0.00715	0.454	0.155
*N*	2750	2750	2750	2750
Log-likelihood	−2553.1	−2480.5	−2421.2	−2414.9
AIC	5122.1	4979.1	4862.5	4851.9
BIC	5169.5	5032.3	4921.7	4917.0

*z statistics in parentheses, errors clustered by respondent id*.

* *p < 0.05*,

** *p < 0.01, and*

*** *p < 0.001*.

*Akaike information criterion (AIC) and Bayesian information criterion (BIC) estimate the relative fit of a particular statistical model for given set of data*.

**Table 4:** Alternative-specific mixed logit specifications, willingness to pay estimates for significant parameters.

**Parameter**	**Specification 1**	**Specification 2**	**Specification 3**	**Specification 4**
Corn Starch	−1.80	−2.34	−1.33	−1.67
Carrageenan	−0.97	−1.02	−0.34	–
MPC	−1.75	−1.71	–	–
Pectin	−0.38	–	–	–
Clean label	–	–	3.53	2.54
Poor texture	–	−1.83	−2.26	−2.83
Clean label × poor texture	–	–	–	1.61

##### Alternative Specific Mixed Logit Model, Texture Control (Specification 2)

Next, we modeled our data using a mixed logit to estimate the impact of price, individual ingredients, and textural defects (poor texture) on the odds of a choice. Inclusion of a dummy variable that controls for poor texture allows for increased resolution into how each stabilizer impacts consumer preferences in this specification by partitioning out the effect of a textural defect. For results, please see estimates for Specification 2 displayed in Table 3. Again, price was found to have a significant negative impact (0.64) on the odds of a choice (*p* < 0.001). When examining the model estimates, we find the impacts of most ingredients are similar to those of our previous model. Consumers are less likely to select yogurts containing carrageenan, MPC, or corn starch. Each ingredient reduces the odds of purchasing a yogurt by 0.63 (carrageenan), 0.46 (MPC), and 0.36 (corn starch), ceteris paribus. However, in this model specification pectin is no longer statistically significant. Based on WTP estimates, consumers are on average willing to pay $1.02 to avoid carrageenan, $1.71 to avoid MPC, and $2.34 to avoid corn starch in yogurt (see Table 4). We introduce a new parameter, poor texture, which allows us to control for yogurts possessing a textural defect. Poor texture has a negative impact on choosing a yogurt with an odds ratio of 0.44 (*p* < 0.001). Therefore, holding all parameters equal, consumers are less likely to purchase a yogurt with a known textural defect relative to a yogurt that where a textural defect not known, and they are willing to pay, on average, $1.83 to avoid a textural defect.

##### Alternative Specific Mixed Logit Model, Texture, and Clean Label Controls (Specification 3)

In our third specification, we model the impact of price, individual ingredients, textural defects, and clean labeling [an ingredient list free of added ingredients (i.e., stabilizers)] on the odds of choosing a yogurt.

The new dummy variable, clean label, controls for clean label ingredient lists (i.e., our baseline ingredient list which contains only cultured pasteurized milk). Inclusion of such a control is important for our analysis as it teases out consumer preference for a clean ingredient list. This allows us to further unravel how each tested ingredient impacts consumer purchase behavior beyond a general preference for a clean, simple ingredient list. This model specification reveals, again, similar impacts for the price (0.58) and poor texture (0.29) (*p* < 0.001). Thus, a textural defect (i.e., poor texture) significantly decreases the odds of purchasing a yogurt, and on average, consumers are willing to pay an average premium of $2.26 to avoid a textural defect. The parameter clean label, which allows us to control for yogurts that have clean ingredient lists, was found to have an odds ratio 6.78 (*p* < 0.001). Therefore, holding all other characteristics equal, consumers are 6.78 times more likely to purchase a yogurt with a clean label than a yogurt without a clean label. Based on its WTP estimate, consumers are willing to pay an average premium of $3.53 for a 32 oz yogurt with a clean label. Looking beyond a clean label, we find that MPC along with pectin are no longer statistically significant (see Table 3). Our results suggest consumers are less likely to purchase a yogurt containing corn starch (0.49, *p* < 0.001) or carrageenan (0.83, *p* < 0.05), and they are, on average, willing to pay an estimated $1.33 and $0.34 to avoid them in yogurt (see Table 4), respectively.

##### Alternative Specific Mixed Logit Model, Texture, and Clean Label Interactions (Specification 4)

Our final mixed logit model specification (Specification 4) examines not only how price, ingredients, clean labels, and texture impact consumer choices for yogurt, but also includes an interaction between clean labels and poor texture. The interaction in this specification allows us to examine whether the impact of a textural defect may depend on whether a yogurt is clean label. Consistent with the results of our previous specifications, we find that that consumers are less likely to purchase a yogurt with a textural defect (0.26, *p* < 0.001; see Table 3) and, on average, are willing to pay $2.83 more to avoid bad texture (see Table 4). The clean label odds ratio is 3.31 for this model, which is relatively large. Thus, based on this model consumers are more likely to purchase a yogurt if it has a clean label, and are willing to pay an average premium of $2.54 for a clean label based on the parameter's WTP estimate. Looking at the interaction, the odds ratio for clean label × poor texture is 2.16 (*p* < 0.001), suggesting that even with a known textural defect, a clean label on a yogurt increases the odds of a purchase. Hence, consumers may be willing to accept a textural defect in a yogurt if it has a clean label, and we estimate that they might be willing to pay an average premium of $1.61 for a clean ingredient list. Lastly, beyond clean labels we find that consumers were less likely to purchase a yogurt containing corn starch (0.46, *p* < 0.001), and they are willing to pay, on average, $1.67 more to avoid it in their yogurt. No other ingredient-specific parameters were found to be statistically significant in this particular model.

##### Latent Class Model

Results of our latent class model fit with two classes are displayed in Table 5. This analysis examines the differential impact of price, individual ingredients, clean labels, and textural defects on the odds of selecting a yogurt for two different consumer groups^3^. Class 1 comprises of ~38% of our sample. For consumers in this class, both price and corn starch were found to have negative impacts on the odds of choosing a yogurt with odds ratios of 0.51 and 0.43, respectively (*p* < 0.001). Consumers in this class are less likely to purchase a yogurt containing corn starch and, on average, are willing to pay $1.26 to avoid it (see WTP estimates in Table 6). Similar to the results of our Model 4 mixed logit, none of the other three stabilizers were found to have a significant impact on the odds of choosing an alternative for Class 1 consumers. Also similar to the results our mixed logits, Class 1 consumers are more likely to purchase a yogurt if it has a clean label and they are willing to pay a premium of $3.81, on average, for a clean ingredient list. The odds ratio for a clean label is 13.08 for these consumers (*p* < 0.001). Segmentation analysis revealed similar results for the impact of textural defects. Consumers are more likely to purchase a yogurt with good texture, odds ratio of 24.05 (*p* < 0.001), compared to yogurts with poor texture, odds ratio of 6.45 (*p* < 0.001). However, odds ratios for both poor and good textures are positive, suggesting that despite the documented importance of sensory qualities in food, consumers are more likely to purchase a yogurt with textural defects rather than opt out of the purchase entirely. Class 1 consumers are willing to pay a premium of $4.72 for a yogurt free of textural defects and $2.77 for a yogurt with a defect. Using the difference between WTP estimates for good and poor texture parameters we can estimate the average premium that consumers are willing to pay to avoid textural defects. Here, Class 1 consumers are willing to pay an average of $1.95 to avoid textural defects in their yogurt.

**Table 5:** Latent class conditional logit model, two classes, alternative specific fixed effects, robust clustered errors.

	**Class 1**	**Class 2**
Price	0.51^***^	0.67^***^
	(−11.59)	(−5.99)
Pectin	0.97	0.85
	(−0.21)	(−1.33)
MPC	0.98	1.21
	(−0.15)	(1.39)
Carrageenan	0.82	0.84
	(−1.33)	(−1.35)
Corn starch	0.43^***^	0.56^***^
	(−5.11)	(−4.48)
Clean label	13.08^***^	5.50^***^
	(9.73)	(6.82)
Good texture	24.05^***^	96.74^***^
	(9.93)	(11.61)
Poor texture	6.45^***^	29.31^***^
	(4.85)	(8.63)
Share of Class 1	0.38^*^	–
	(2.21)	–
*N*	8,250	
Log-likelihood	−2240.8	
AIC	4515.6	
BIC	4634.9	

*Odds ratios are displayed. z statistics in parentheses, errors clustered by respondent id*.

* *p < 0.05 and*

*** *p < 0.001*.

*Akaike information criterion (AIC) and Bayesian information criterion (BIC) estimate the relative fit of a particular statistical model for given set of data*.

**Table 6:** Latent class logit willingness to pay estimates for significant parameters.

**Parameter**	**Class 1**	**Class 2**
Corn starch	−1.26	−1.42
Clean label	3.81	4.22
Estimated premium for good texture^a^	1.95	2.96

a *Calculated from the difference of WTP for Good Texture and WTP for Poor Texture (Good texture – poor texture)*.

The remaining and larger portion of our sample (62%) belongs to Class 2. Similar to Class 1, both price and corn starch were found to have negative impacts on the odds of selecting an alternative with odds ratios of 0.67 and 0.56, respectively (*p* < 0.001; see Table 5). WTP estimates reveal that, on average, consumers in this class are willing to pay a premium of $1.42 to avoid corn starch in yogurt (see Table 6). The odds ratio for clean label was 5.50 (*p* < 0.001), thus clean labels are also important for consumers in this class and they are, on average, willing to pay a premium of $4.22 for a yogurt with a clean ingredient list. Considering the texture parameters for Class 2, the odds ratios for good and poor texture were 96.74 and 29.3, respectively. The textural attribute WTP estimates for this class reveal that Class 2 consumers are willing to pay a premium of $11.32 for a yogurt free of textural defects and $8.36 for a yogurt with a defect. In line with Class 1, consumers in Class 2 would rather purchase a yogurt with some level of textural defect rather than not purchase a yogurt at all. However, to avoid textural defects, consumers in Class 2 are willing to pay an average premium of $2.96.

## Discussion

Previous research has supported that naturalness (84) and food qualities (27–29) are important to consumers. Others have shown that labeled attributes (i.e., organic) can also impact consumers' perception of product quality (42, 85). However, to best of our knowledge, this study is the first to examine the interactive impacts of ingredients and potential quality (textural) defects on consumer choice of foods. Additionally, the current study evaluates the impact of ingredients, considered both “clean” and conventional, on willingness to pay for yogurt. Specifically, we examined the demand for clean labeled plain yogurt using an a computerized in-person choice experiment. Our experimental design included 35 hypothetical yogurts presented in 11 choice sets consisting of two yogurt options, and an opt out “no purchase” option in each choice set. Our results summarized in this paper reveal some interesting findings.

First, we find that clean label (i.e., ingredient list lacking added stabilizers and/or thickening agents) increase the odds of choosing a plain yogurt compared to those containing one or more stabilizers. For 32 oz family size plain yogurt, the average willingness to pay premium for a clean label is between $2.54 and $3.53. Our analysis consisted of four mixed logit model specifications that increased in complexity. In our base specification (Specification 1), all four stabilizers were found to have a significant, negative impact on consumer choice. However, as we added parameters to tease out the impact of a clean label ingredient list and textural defects in our model using a step-wise approach, we found different revelations in consumer preferences for the ingredients we tested. Our most exhaustive specification (Specification 4) revealed that, beyond a clean label, corn starch was the only ingredient tested that consumers may be specifically avoiding in plain yogurts, beyond in general looking for a label with a minimum number of ingredients. This finding may be worth considering for yogurt companies that currently produce plain yogurts with corn starch, particularly for those looking to clean up their labels. We estimate that on average consumers may be willing to pay between additional $1.33 and $2.34 (per 32 oz) to avoid corn starch on a yogurt label. Maruyama et al. (52) reported that corn starch was rated as more natural compared to both MPC and carrageenan by consumers. The combined findings reported here and the previous results of Maruyama et al. (52) suggest that while corn starch may be perceived as a relatively natural and familiar ingredient in general, its presence may not be considered acceptable in the context of plain yogurt. Consumers may consider a yogurt that requires thickening to be of lower quality, resulting in the observed lower WTP. As other thickeners and their functions are less familiar to consumers (52), they are avoided as part of the demand for a minimal ingredient label, rather than avoided specifically. The exact mechanism and the potential explanations for the observed behavior around corn starch in yogurt warrant further investigation. It is worthwhile to note that these findings may not directly apply to flavored yogurts. Additional research focusing on consumer demand for clean labeled flavored yogurts is recommended.

Second, this study found that a textural defect decreases the odds of purchasing a yogurt. Based on our mixed logit analysis, the average premium to avoid a textural defect may fall between $1.83 and $2.83. While segmentation analysis did not reveal much variation in consumer preferences, it suggested that consumers are more likely to purchase a yogurt with a textural defect rather than opt out of a purchase entirely. One caveat with respect to the impact of textural defects is the limited description of the defect presented in our experiment. In our study, the only textural defect examples were the descriptors “grainy” and “lumpy” [the full description was given as “Consumers generally perceive the texture of this yogurt to have some defects (e.g., grainy, lumpy)]”. These descriptors were presented together on a single attribute level, thus we cannot untangle whether one defect (i.e., grainy) is more impactful than the other (i.e., lumpy). It is possible that consumers would respond differently to these defects as well as some others. Examples of other yogurt textural defects include weak and firm/gel-like yogurt body (86). A weak body defect is characterized by a runny, liquid-like texture. On the other hand, a firm/gel-like defect is characterized by excessive firmness, which impacts how easily a yogurt compresses and melts away in the mouth during consumption. We recommend further research that examines the specific impacts of types of textural defects on consumer acceptance and their willingness to pay for clean label yogurt.

Third, we examined the impact of textural defects in relation to a clean ingredient list. The interaction included in the fourth mixed logit model specification revealed that a clean label on a yogurt might temper the negative impact of a textural defect. This finding may offer reassurance to companies that have not been successful in producing clean label yogurts with ideal texture; consumers may actually be willing to accept a less than ideal texture in yogurts with clean ingredient lists. We estimate the average willingness to pay for a clean label yogurt with a poor texture to be an average premium of $1.61.

Additional insights into consumer preferences were collected from our respondents in a questionnaire that was administered directly following the choice experiment. Recall, participants rated their agreement on a series of different statements designed to capture the importance of various yogurt-related attributes (see Table 2). A 10-point scale anchored at 1 indicating strong disagreement and 10 indicating strong agreement with each statement presented was used. This allowed us to capture the average stated preference of the attributes tested. On average, the highest rated attribute was yogurt quality, defined as characteristics such as taste and texture. Quality was followed by price and ingredients. Of note, the attribute “ingredients” refers to ingredients in general and does not specify the ingredients (i.e., stabilizers) tested in our study. Interestingly, while ingredients were rated as being relatively important to consumers, the presence of corn starch, carrageenan, MPC, or pectin were not rated to be very important. These seemingly conflicting findings in our questionnaire demonstrate some of the limitations of stated preferences studies. While choice experiments utilize stated preferences, elicitation of preferences through our experiment allowed us to examine the impact of these attributes in the context of an ingredient list and in the presence or absence of textural defects. In doing so we find that, in the case of plain yogurt, having a clean label matters and consumers are willing to accept some level of textural defects in a clean label yogurt.

## Conclusions

Price and quality are important attributes for consumers, but in our choice experiment, consumers displayed a clear preference for clean labels, specifically, a minimal ingredient list. The results of our hypothetical choice experiment reveal that, on average, consumers may be willing to pay between $2.54 and $3.53 for a clean label on a plain 32-ounce container of yogurt, and the negative impact of textural defects can be attenuated by the positive effect of a clean label in our study. Our latent class modeling revealed two consumers classes with similar preference patterns that would, on average, prefer to purchase yogurts with textural defects rather than opt out of purchasing yogurt entirely.

Altogether, this presents important implications for policy makers that are considering introducing policies reducing producers' ability to use particular ingredients (due to health or environmental concerns): consumers are willing to take some textural deficiencies in return for a cleaner label. This is good news for the food manufacturers as well, as reformulation of food products is often associated with performance challenges.

Additionally, our results suggest that a marginal approach to cleaning up a food label might not be appreciated by consumers for all ingredients. Specifically, our results suggest that removing just one thickener out of a longer ingredient list would only have a positive impact on consumer demand in case of starch, and not lead to an appreciable increase of consumer WTP for other ingredients. Our results are a step in the direction of examining the complex issues of clean labels, product sensory performance, and consumer demand. However, the use of hypothetical scenarios can lead to hypothetical bias on respondent choices (87), highlighting a limitation of the study. Hypothetical bias often results in inflated estimates of willingness to pay in stated preference valuation studies like a choice experiment (88). To mitigate bias, a cheap talk script was delivered to each respondent prior to starting the experiment. Cheap talk scripts have been used as a tool for mitigating bias in choice experiments (89) and have been shown effective in obtaining more reliable estimates (90). As a next step in examining the impact of varying ingredients and sensory characteristics on consumer preferences, we recommend a revealed preference valuation study such as an experimental auction, where consumers can actually taste yogurts and evaluate textural defects prior to placing a bid and potentially parting with money for real products. Considerations for such an auction would be procuring or making yogurts with ingredient lists and sensory attributes of interest.

## Data Availability Statement

The raw data supporting the conclusions of this article will be made available by the authors, without undue reservation.

## Ethics Statement

The studies involving human participants were reviewed and approved by Oregon State University IRB board. The patients/participants provided their written informed consent to participate in this study.

## Author Contributions

SM, NS, and JL contributed to conception and design of the study. SM and NS performed the statistical analysis and wrote the first draft of the manuscript. JL wrote sections of the manuscript. All authors contributed to manuscript revisions, read, and approved the submitted version.

## Conflict of Interest

The authors declare that the research was conducted in the absence of any commercial or financial relationships that could be construed as a potential conflict of interest.

## Publisher's Note

All claims expressed in this article are solely those of the authors and do not necessarily represent those of their affiliated organizations, or those of the publisher, the editors and the reviewers. Any product that may be evaluated in this article, or claim that may be made by its manufacturer, is not guaranteed or endorsed by the publisher.

## Footnotes

^1^Carrageenan is a stabilizer that is only used when combined with other stabilizers or thickening agents in yogurt formulations. While it is not used as a standalone stabilizer, it was presented as one in some of our choice sets as this was needed to determine its impact on consumer preferences.

^2^One choice set displayed a price of $4.36 instead of $4.49. Observations that responded to this particular choice set were dropped and data analysis was re-run. Results from this subsequent analysis produced similar results.

^3^For this analysis, we have used a categorical, rather than a dummy, variable for texture due to coding differences of the estimation routines for latent class and alternative specific mixed logit models.
